# Ginsenoside Rg3 Inhibits Melanoma Cell Proliferation through Down-Regulation of Histone Deacetylase 3 (HDAC3) and Increase of p53 Acetylation

**DOI:** 10.1371/journal.pone.0115401

**Published:** 2014-12-18

**Authors:** Xiu Shan, Yuan-Shan Fu, Faisal Aziz, Xiao-Qi Wang, Qiu Yan, Ji-Wei Liu

**Affiliations:** 1 Department of Oncology, the First Affiliated Hospital of Dalian Medical University, Dalian, Liaoning Province, China; 2 Department of Human Anatomy, Dalian Medical University, Dalian, Liaoning Province, China; 3 Department of Biochemistry and Molecular Biology, Liaoning Provincial Core Lab of Glycobiology and Glycoengineering, Dalian Medical University, Dalian, Liaoning Province, China; 4 Department of Dermatology, Northwestern University Feinberg School of Medicine, Chicago, Illinois, United States of America; Wayne State University School of Medicine, United States of America

## Abstract

Malignant melanoma is an aggressive and deadly form of skin cancer, and despite recent advances in available therapies, is still lacking in completely effective treatments. Rg3, a monomer extracted from ginseng roots, has been attempted for the treatment of many cancers. It is reported that the expressions of histone deacetylase 3 (HDAC3) and p53 acetylation correlate with tumor cell growth. However, the antitumor effect of Rg3 on melanoma and the mechanism by which it regulates HDAC3 expression and p53 acetylation remain unknown. We found high expression of HDAC3 in human melanoma tissues to be significantly correlated to lymph node metastasis and clinical stage of disease (*p*<0.05). In melanoma cells, Rg3 inhibited cell proliferation and induced G0/G1 cell cycle arrest. Rg3 also decreased the expression of HDAC3 and increased the acetylation of p53 on lysine (k373/k382). Moreover, suppression of HDAC3 by either siRNA or a potent HDAC3 inhibitor (MS-275) inhibited cell proliferation, increased p53 acetylation and transcription activity. In A375 melanoma xenograft studies, we demonstrated that Rg3 and HDAC3 short hairpin RNA (shHDAC3) inhibited the growth of xenograft tumors with down-regulation of HDAC3 expression and up-regulation of p53 acetylation. In conclusion, Rg3 has antiproliferative activity against melanoma by decreasing HDAC3 and increasing acetylation of p53 both *in vitro* and *in vivo*. Thus, Rg3 serves as a potential therapeutic agent for the treatment of melanoma.

## Introduction

Melanoma is a deadly form of skin cancer. The incidence of melanoma has increased worldwide, and its mortality rate continues to rise faster than most other forms of cancers [1], [2]. The short-lived therapeutic response, tumor resistance, and adverse effects of traditional drugs used in melanoma therapy, including dacarbazine, temozolomide and interleukin-2, along with recently approved targeted therapeutic agents, such as vemurafenib and ipilimumab, have limited their use due to low responsive rates and/or high toxicities [3]–[5]. Therefore, the development of novel therapeutic strategies and targets are needed for the treatment of melanoma.

Acetylation and deacetylation are epigenetic processes that are in the regulation of gene expression. Histone acetylation catalyzed by histone acetyltransferases (HATs) is typically associated with inducing gene transcription, while histone deacetylation controlled by histone deacetylases (HDACs) is mainly related to gene silence [6]. Until now, 18 HDACs have been identified in HDACs family, and they are generally divided into four classes based on the homology of sequence to yeast counterparts. Class I contains HDAC 1, 2, 3, 8 which show homology to the yeast HDAC, Rpd3; class II contains HDAC 4, 5, 6, 7, 9, 10 which have a high degree of homology to yeast Hda1 gene; class III contains the homologous to the yeast Sir2 (SIRT1-7); and class IV contains HDAC11, which has some common features of HDACs in both class I and II. The abnormal expression of HDACs has been found in various types of cancer. For instance, HDAC6 is highly expressed in human pancreatic and breast cancers [7], [8], overexpression of HDAC2 has been found in cervical and gastric cancers [9], [10], and high levels of HDAC8 expression have been reported in childhood neuroblastoma [11]. Overexpression of HDAC3 is reported in many cancer types, such as lung, colorectal and gastric cancer, as well as in prostate cancer [12]–[14]. The function of HDAC3 is not only as a corepressor for many sequence-specific transcription factors (E2F/Rb, NF-kB, c-jun), but it also binds to specific promoters and regulates transcription through deacetylation of histones or non-histones substrates. p53 is the first non-histone protein which can be deacetylated by the specific HDACs [15]. For example, HDAC1 interacts with p53 and deacetylates it *in vivo* and *in vitro* [16]. HDAC3 shares the high homology with HDAC1, hence we hypothesized that HDAC3 deacetylates p53, and leads to increase of tumor cell growth.

Previous studies suggested that abnormal expression of HDACs was associated with tumor growth, invasion, and metastasis [14], [17], [18]. Therefore, HDACs have emerged as the important therapeutic targets for many cancers. HDAC inhibitors (HDACis) have the ability to decrease cell proliferation, promote differentiation, induce apoptosis, and inhibit angiogenesis of cancer through preventing the deacetylation of histones and non-histones [19]. According to their structures, HDACis can be classified into six groups, such as hydroximates (e.g., TSA, vorinostat/SAHA), cyclic peptides (e.g., romidepsin), benzamides (e.g., entinostat/MS-275), and so on. Currently, HDACis have already been approved by the FDA for the treatment of cutaneous T-cell lymphoma (vorinostat and romidepsin) and peripheral T-cell lymphoma (belinostat) [20], [21]. Several clinical trials evaluating the efficacy of HDACis in other forms of cancers are ongoing [22].

Ginsenosides, as found in traditional Chinese medicine, have been reported to exhibit antitumor properties in some cancers, such as malignant hepatic, gastric, and prostate tumors [23]–[25]. However, there are no data about the effects of Rg3 on human melanoma. 20 (R)-Rg3 is a monomer extracted from ginseng, and it has been found to have antitumor effects include anti-proliferation, anti-metastasis, anti-angiogenesis, enhancing chemotherapeutic susceptibility, as well as immune stimulation. Shen Yi capsule which contains Rg3 as the main ingredient has been approved by China Food and Drug Administration (CFDA) to be used clinically for cancer treatment [26]. However, the interplay between Rg3, HDAC3, and melanoma growth remains unclear.

In this study, we demonstrated the antitumor effect of Rg3 against melanoma both *in vitro* and *in vivo*. Moreover, the antitumor mechanism of Rg3 was correlated to down-regulation of HDAC3 expression and increased p53 acetylation.

## Materials and Methods

### Ethics Statement

This study was approved by the Institutional Review Boards (IRBs) of Dalian Medical University, China. We collected paraffin sections of patients from the First Affiliated Hospital of Dalian Medical University. We used the patients' tissues in a retrospective study. After carrying out telephone interviews, verbal consent for the use of the tissues of patients was obtained from them or their next of kin. This consent procedure was approved by our IRB. The investigation was conducted in accordance with humane and ethical research principles of Dalian Medical University.

All animal work performed in this study was approved by the Animal Ethics Committee of Dalian Medical University. Moreover, the detail protocols and experimental processes were conformed to the Experimental Animal Management Regulations of Dalian Medical University (Permit Number: #3555).

### Reagents and cell cultures

20 (R)-Rg3 was provided by Dalian Fu Sheng Pharmaceutical Company (Dalian, China) and diluted freshly in Dulbecco's Modified Eagle's Medium (DMEM) (Invitrogen, Carlsbad, CA) (500 µg/mL) and filtered through 0.22 µm sterile membrane. It was diluted with cell culture media to the final concentrations for different treatments. MS-275 was purchased from Selleck Chemicals (USA) and dissolved in culture-grade DMSO (Sigma-Aldrich, St. Louis, MO, USA) before diluted in a complete medium to its working concentration. The p53 promoter-luciferase reporter plasmid was constructed in TransGen Biotech Company (Beijing, China). The p53 wild type (wt) melanoma cells A375 (original commercial source from American Type Cell Culture, ATCC, Manassas, VA) and C8161 [27] were kindly provided by Dr. XQ Wang (Department of Dermatology, Northwestern University Feinberg School of Medicine, USA). The p53 mutant melanoma cells SK-MEL-28 were purchased from ATCC. All cell lines were cultured in DMEM supplemented with 10% fetal bovine serum (FBS, Invitrogen), 100 U/mL penicillin, and 50 µg/mL streptomycin at 37 °C under 5% CO_2_ in humidified air.

### Patients and clinical tissue specimens

Patients enrolled in this study were diagnosed with histopathologically confirmed melanoma from 2003 to 2010. Among these patients, 32 patients were found to be eligible for clinical evaluation. All specimens were fixed in formalin and embedded in paraffin at the department of pathology at the First Affiliated Hospital, Dalian Medical University. Tumors were staged according to the American Joint Committee on Cancer (AJCC) classification (2009).

### Cell viability assay

Melanoma cells were plated at a density of 2,000 cells/well in 96-well plates. The cytotoxic effect of drugs was evaluated in cells cultured for 24, 48 or 72 h using cell counting assay kit-8 (CCK-8) according to the manufacturer's instructions (Dojindo Laboratories, Japan). Briefly, 10 µL of the CCK-8 solution was added to cell cultures for the designated times. Plates were incubated for 2 h at 37 °C. CCK-8 solution was reduced by dehydrogenases in cells to yield an orange-colored product (formazan), which was soluble in the tissue culture medium. The amount of the formazan dye (450 nm absorbance) generated by dehydrogenases in the cells was directly proportional to the number of living cells.

### Construction of RNA interference sequences and transfection

Two HDAC3-specific small interference RNA (siRNA) sequences were as follows, siRNA-1: 5′-GAGCUUCCCUAUAGUGAAUTT-3′, 5′-AUUCACUAUAGGGAAGC UCTT-3′; siRNA-2∶5′-CCGCCAGACAAUCUUUGAATT-3′, 5′-UUCAAAGAUUGUCUGGCGGTT-3′. One HDAC3 short hairpin RNA sequence was as follows, shRNA: 5′-GAGCUUCCCUAUAGUGAAUTT-3′, 5′-AUUCACUAUAGGGAAGCUCTT-3′. All sequences were purchased from the GenePharma Company (Shanghai, China). Melanoma cells were seeded onto six-well plates. When cells reached 60–70% confluence, HDAC3 siRNA was transiently transfected into the cells using Lipofectamine 2000 Reagent (Invitrogen) according to manufacturer's instructions. The transfection reagent was removed after 5 h and the cells were harvested after 48 h.

### Quantitative Real-time PCR analysis

Total RNA was extracted using TRIzol (Invitrogen) according to the manufacturer's protocol. RNA was reverse transcribed into cDNA using PrimeScript^TM^RT reagent kit (Takara, Japan). The HDAC3 primers were 5′-CACCCTATGAAGCCCCATCG-3′ (Forward), 5′-GAGACCGTAATGCAGGACCAG-3′ (Reverse); the PCNA primers were 5′-GGCCGAAGATAACGCGGATAC-3′ (Forward), 5′-GGCATATACGTGCAAATTCACCA-3′ (Reverse); the GAPDH primers were 5′-ATGGGGAAGGTGAAGGTCG-3′ (Forward), 5′-GGGGTCATTGATGGCAACAATA-3′ (Reverse). Real-time quantitative PCR reaction was carried out in a Thermal Cycler Dice system (Takara, Japan). Relative HDAC3 and PCNA mRNA levels were normalized with GAPDH and calculated using 2*^−ΔΔCT^* method.

### Western blotting

Cells were washed with PBS (pH 7.4), and incubated with 2× concentrated electrophoresis sample buffer (125 mM Tris-HCl, pH 6.8, 5% glycerol, 2% SDS, 1% β-mercaptoethanol) for 30 min on ice. Protein concentration was determined with Coomassie protein assay reagent using bovine serum albumin as a standard. Total protein (50–70 µg/lane) from the whole cell lysates was separated by 12% SDS-PAGE and proteins separated in the gel were transferred electrophoretically onto nitrocellulose membrane (Millipore Billerica, MA, USA). Antibodies against HDAC3, Ac-p53 (k382) (1∶1000) were obtained from Abcam (Cambridge, UK). Ac-p53 (k373) (1∶1000) was obtained from Upstate-Millipore (Massachusetts, USA). p53 and p21 (1∶200) were obtained from BD (Pharmingen, USA). PCNA, PRB, cyclin E, cyclin D1, CDK4, CDK2 and GAPDH antibodies (1∶500) and HRP-conjugated anti-rabbit or anti-mouse antibody (1∶2000) were obtained from Proteintech Group (Chicago, IL, USA). ECL (enhanced chemiluminescence) detection system (Bio-Rad) was used to visualize immunoreactive bands.

### Immunofluorescent staining

After washing with PBS, cells grown on coverslips were fixed with 4% paraformaldehyde for 30 min. Cells were then permeabilized with 0.1% Triton X-100 for 5 min. After being blocked with 3% serum for 30 min at 37 °C, cells were incubated with rabbit anti-HDAC3 antibody (1∶200) or Ac-p53 (k373/k382) (1∶200) at 4 °C overnight. The cells were then incubated with FITC or TRITC-conjugated goat anti-rabbit IgG (1∶200) (Sigma-Aldrich, St. Louis, MO) for 1 h. Images were captured with the Olympus BX83 fluorescence microscope (Japan).

### Cell cycle analysis

Cells were cultured in 6 cm dishes and allowed to grow to 75–80% confluent. Collected cells were fixed with ethanol and stained with propidiumiodide in PBS. The cell suspension was incubated in the dark for 30 min at room temperature and subsequently measurement in a FACScan flow cytometer (BD Biosciences). The percentages of cells at the G0/G1, S, and G2/M phases were obtained from three independent experiments.

### Luciferase reporter assay

p53 luciferase activity was determined in A375 and C8161 cells cotransfected, using Lipofectamine 2000 Reagent, with 2 µg of p53 luciferase plasmids and 0.2 µg of pGL3, which constitutively expressed renilla luciferase. Twenty-four hours post transfection, the cells were treated with Rg3 (50 µg/mL) or MS-275 (10 µM) for 24 h, or treated with siHDAC3 for 24 h. The luciferase activity was assayed using the Dual Luciferase Reporter Assay System (Berthold technology, Germany). Firefly luciferase activity was measured and the reading was normalized to renilla luciferase activity, which served as an internal control for transfection efficiency.

### Xenograft tumor models of human melanoma

Male nude mice (Balb/c-nu/nu) were obtained from Animal Center (Dalian Medical University). The animals (4–6 weeks) were maintained under sterile conditions during the entire experimental period. A375 cells (2×10^6^) suspended in 0.2 mL PBS were injected subcutaneously into the right flank. After one week of tumor cell injection, tumor-bearing mice were randomly divided into various treatment and control groups (n = 6 per group). Rg3 was administered at 20 mg/kg body weight to mice 5 times per week for 3 weeks via intraperitoneal injection; shHDAC3 and vector (150 µg DNA/mouse) were injected at the tumor cell inoculation sites, and the treatment was repeated every 48 h; combination of Rg3 at 20 mg/kg and shHDAC3 at 150 µg DNA/mouse was each administered by subcutaneous and intraperitoneal injection 4 times per week for 3 weeks. The control groups received the vehicle or vector only. Tumor volume was measured by Vernier calipers every other day after tumor inoculation. The tumor volume was computed according to the formula 1/2 a × b^2^, where ‘a’ was the length of the long axis and ‘b’ was the length of the short axis (mm). All animal protocols were conformed to the Experimental Animal Management Regulations of Dalian Medical University.

### Immunohistochemical staining in human tissues and xenograft tumors

Paraffin sections (4 µm) of patient tissues and mouse xenograft tumors were either stained with H & E or immunostained using DAB as a substrate. For xenograft tumors, each excised tumor tissue was fixed in 5% formalin for 24 h before embedded in paraffin and processed sectioning. For immunohistochemical analysis of HDAC3 and Ac-p53 (k373/k382) expression, the slides were deparaffinized and rehydrated using standard techniques. Non-specific binding sites were blocked with complete serum at 37 °C for 30 min. Then, the tissue sections were incubated with the HDAC3 or Ac-p53 (k373/k382) antibody (1∶200) at 4°C overnight. The signal was visualized with peroxidase-labeled streptavidin-complexed DAB, and the sections were briefly counterstained with hematoxylin. Yellowish-brown stain indicated a positive result. The negative control was generated by replacing the primary antibody with PBS. Images were captured with the Olympus BX51 microscope (Japan). We graded the staining intensity as follows: negative (score 0), 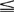
25% (score 1), 26–50% (score 2), 51–75% (score 3) and 76–100% (score 4). The immunohistochemical staining was evaluated and scored by at least two independent pathologists.

### Statistical analysis

All data were expressed as means±standard deviation (SD). The correlation between HDAC3 expression and each clinical feature was evaluated by Fisher's exact test. Other data were analyzed statistically by two-tailed *Student*'*s t* test. *p*<0.05 was considered to be significant and *p*<0.01 was considered to be highly significant.

## Results

### Increased expression of HDAC3 correlates with lymph node metastasis and clinical stage of melanoma

**Figure 1:**
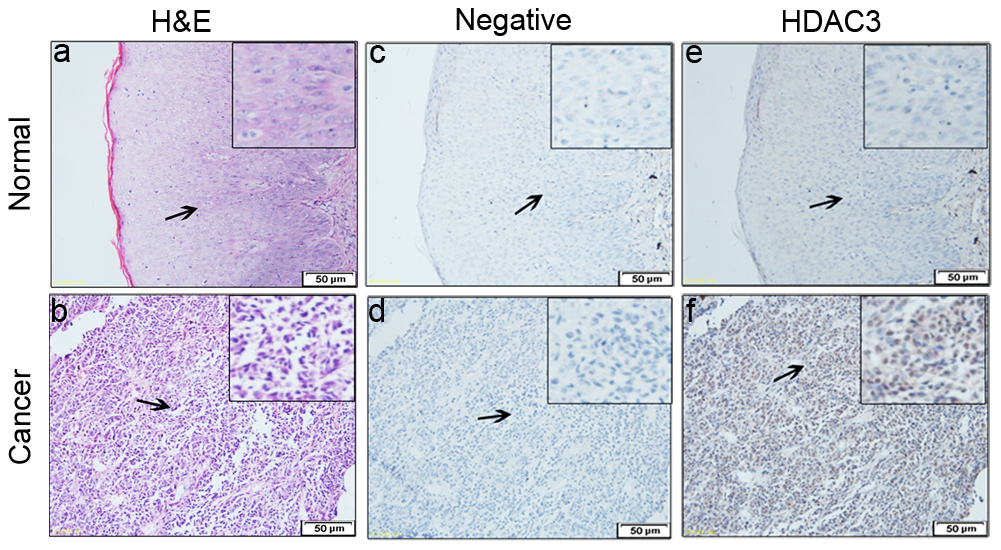
Increased expression of HDAC3 correlates with lymph node metastasis and clinical stage of melanoma. Immunohistochemistry staining of HDAC3 in human melanoma tissues. Normal and cancer tissues were analyzed by H&E staining (a, b). Negative control of normal and cancer tissues (c, d). The expression of HDAC3 in normal and cancer tissues (e, f). Bar = 50 µm (magnification, ×200). Insets showed enlarged views (magnification, ×400).

We used an immunohistochemical approach to analyze HDAC3 expression in melanoma patients' specimens compared to that of normal tissues. Results showed that HDAC3 was highly expressed in melanoma samples, particularly among the tumor-invaded zones, whereas non-melanoma samples showed low expression (**Fig. 1**). The clinicopathologic features (**Table 1**) demonstrated that a high proportion of melanoma specimens (23/32, 72.0%) stained strongly for HDAC3 (score 3–4). The results also suggest that there are no significant differences in HDAC3 staining based on age, sex, melanoma subtype, and melanoma ulceration, but high HDAC3 level is correlated with increased lymph node metastasis and clinical stage of melanoma (*p*<0.05).

**Table 1:** Correlation of HDAC3 protein expression to clinicopathologic features of melanoma Patients.

	N = 32		HDAC3		
Clinicopathologic factor	No.	%	High staining (score 3–4)	Low staining (score 1–2)	*P-*value
Age at diagnosis (years)					
≤65	19	59.4	16	3	0.109
> 65	13	40.6	7	6	
Sex					
Male	17	53.1	13	4	0.699
Female	15	46.9	10	5	
Tumor subtype					None
Acral	15	46.9	8	7	
Mucosal	4	12.5	4	0	
CSD	4	12.5	3	1	
NSD	6	18.8	5	1	
UP	3	9.3	3	0	
Ulceration					
Yes	18	56.2	13	5	1.000
No	14	43.8	10	4	
Lymph node metastasis					
Absent	15	46.9	8	7	0.049
Present	17	53.1	15	2	
Stages					
I–II	13	40.6	6	7	0.015
III–IV	19	59.4	17	2	

Abbreviations: CSD, melanomas on skin with chronic sun-induced damage;NSD, melanomas on skin without chronic sun-induced damage; UP, melanoma of unknown primary.

### Rg3 inhibits melanoma cell growth

**Figure 2:**
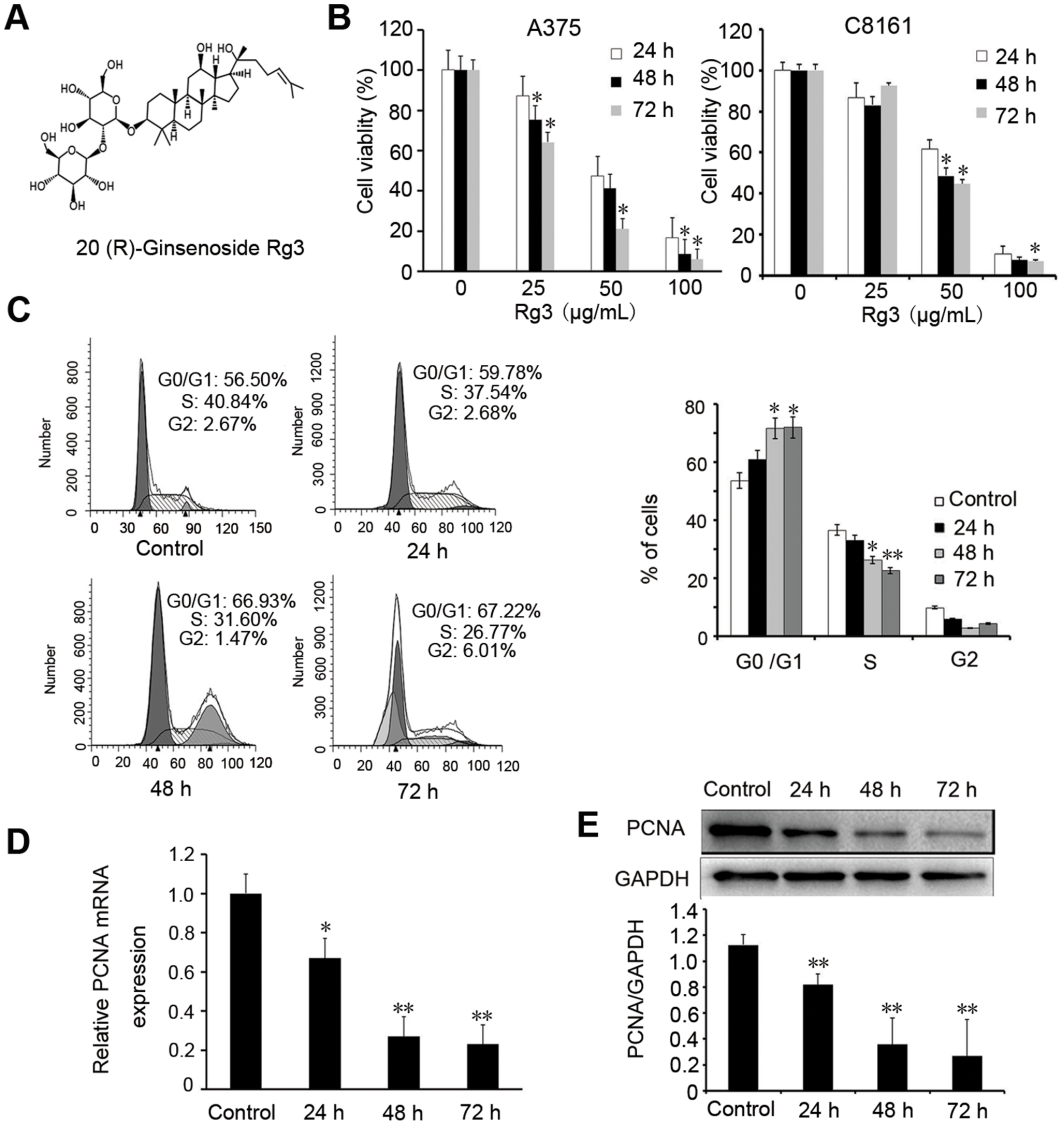
Rg3 inhibits melanoma cell growth. (A) Structure of 20 (R)-Ginsenoside Rg3. (B) A375 and C8161 cells were treated with Rg3 (0, 25, 50, 100 µg/mL) for 24, 48 or 72 h. Cell viability was determined by CCK-8 assay as described in Materials and Methods. (C) Cell cycle analysis. A375 cells were treated with Rg3 (50 µg/mL) for 0, 24, 48 or 72 h and were analyzed by a FACScan flow cytometer. Group data analysis was shown. (D) The mRNA level of PCNA was detected by real-time PCR in A375 cells. (E) Western blotting was employed to examine PCNA protein expression in A375 cells. GAPDH was used as an internal control. The statistical analyses for Western blotting were shown. Data were presented as the mean±S.D. of three independent experiments. *, *p*<0.05; **, *p*<0.01.

To evaluate the antiproliferative effects of Rg3 (structure shown in **Fig. 2A**) on melanoma *in vitro*, we treated A375 and C8161 cells with different concentrations of Rg3 (0, 25, 50, 100 µg/mL) for 24 h, 48 h, and 72 h. The CCK-8 proliferation assay showed the growth inhibition in a time- and dose-dependent manner (**Fig. 2B**), with IC_50_ values of 50.23 µg/mL at 24 h, 42.31 µg/mL at 48 h, 31.8 µg/mL at 72 h in A375 cells, and 55.63 µg/mL at 24 h, 50.26 µg/mL at 48 h, 49.40 µg/mL at 72 h in C8161 cells. The viabilities of A375 and C8161 cells were significantly decreased after treatment with 50 or 100 µg/mL of Rg3 (*p*<0.05). Flow cytometry was used to detect the effect of Rg3 on cell cycle distribution in A375 cells. As shown in **Fig. 2C**, Rg3 treatment at 50 µg/mL arrested most cells at G0/G1 phases and significantly reduced cells at S phase in a time-dependent manner (*p*<0.05). To further confirm the antiproliferative effects of Rg3 on melanoma cell growth, the PCNA level, a commonly used marker to evaluate cell proliferation, was examined. PCNA expression, as determined by real-time PCR (**Fig. 2D**) and Western blotting **(** **Fig. 2E** **),** was significantly decreased in Rg3 treatment A375 cells.

### Rg3 decreases HDAC3 expression

**Figure 3:**
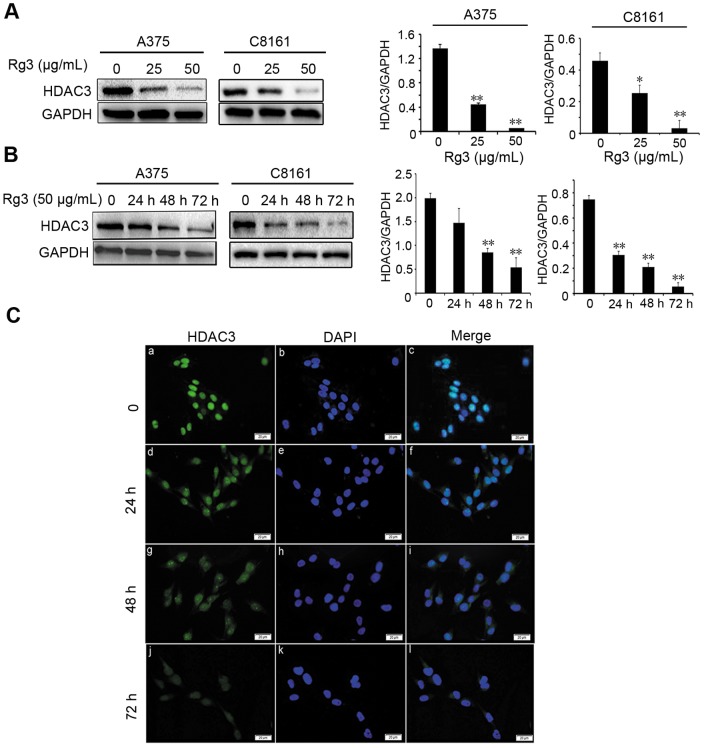
Rg3 decreases HDAC3 expression. (A) Analysis of HDAC3 level by Western blotting in A375 and C8161 cells after cells treated with Rg3 (0, 25, 50 µg/mL) for 72 h. (B) HDAC3 expression level in cells treated with Rg3 (50 µg/mL) for 0, 24, 48 or 72 h. GAPDH was used as an internal control. The statistical analyses for Western blotting results were shown (*, *p<*0.05; **, p<0.01). (C) Immunofluorescent staining of HDAC3 in A375 cells after Rg3 (50 µg/mL) treatment for 0, 24, 48 or 72 h. DAPI used as a nuclear staining. Bar  = 20 µm (magnification, ×400). Data were presented as the mean±S.D. of three independent experiments.

The inhibitory roles of Rg3 on the expression of HDAC3 were examined in A375 and C8161 cells. Western blotting results showed that high concentration of Rg3 treatment (50 µg/mL) inhibited HDAC3 expression, compared to low dose (25 µg/mL) treatment and the control (**Fig. 3A**). We also found that HDAC3 expression was significantly decreased in the cells treated with Rg3 for 72 h, compared to shorter treatment times (**Fig. 3B**). Immunofluorescent analysis of HDAC3 in A375 cells showed similar results as above, with the weakest HDAC3 staining in cells treated with a high dose of Rg3 (50 µg/mL) for the longest experiment time (72 h) (**Fig. 3C**). In summary, Rg3 treatment inhibited the HDAC3 expression in a dose- and time-dependent manner. The data suggests that HDAC3 is a potential target of Rg3.

### Down-regulating HDAC3 expression inhibits melanoma cell proliferation

**Figure 4:**
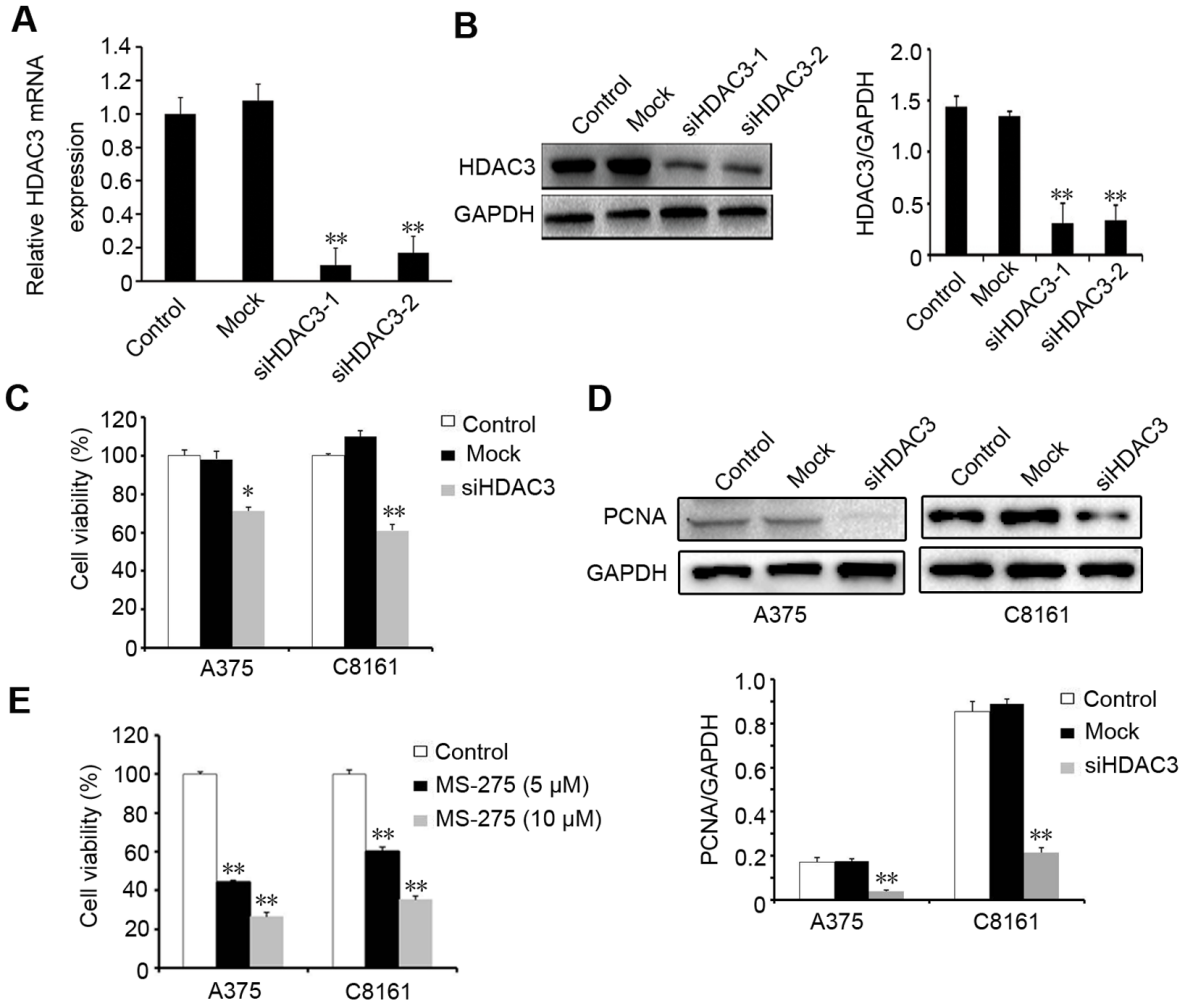
Down-regulating HDAC3 expression inhibits melanoma cell proliferation. HDAC3 was detected by real-time PCR (A) and Western blotting (B) in A375 cells after tansfection with HDAC3 siRNAs. Cell viability was determined by CCK-8 assay in A375 and C8161 cells (C). PCNA protein expression in A375 and C8161 cells (D). Control: untransfected cells; Mock: cells transfected with vector; siHDAC3: cells transfected with HDAC3-1 or HDAC3-2 siRNA. Cells were exposed to MS-275 (5 µM, 10 µM) for 24 h. Cell viability was determined by CCK-8 assay (E). GAPDH was used as an internal control. The statistical analyses for Western blotting results were shown Data were presented as the mean±S.D. of three independent experiments. *, *P*<0.05; **, *P*<0.01.

To investigate the potential relationship between HDAC3 and cell proliferation, we developed two siRNA interference sequences (siHDAC3-1, siHDAC3-2) to silence HDAC3 expression in A375 cells. The results showed that HDAC3 expression was suppressed both in mRNA (**Fig. 4A**) and protein (**Fig. 4B**) levels in siRNA transfected cells, compared to the untreated control and mock transfected cells. Furthermore, CCK-8 assay indicated that cell viability in HDAC3 knockdown cells was significantly decreased in comparison to the untreated control and mock transfected A375 and C8161 cells (**Fig. 4C**). The decreased PCNA expression by Western blotting **(** **Fig. 4D** **)** confirmed the antiproliferative potential after down-regulating HDAC3 level. The potent HDAC3 inhibitor (MS-275) was also tested with regards to melanoma cell proliferation, and also showed an antiproliferative effect. Cell viability in MS-275 treated cells was significantly decreased (*p*<0.05) in comparison to untreated A375 and C8161 cells (**Fig. 4E**). These results demonstrate that down-regulating HDAC3 expression can inhibit melanoma cell proliferation.

### Rg3 and down-regulating HDAC3 expression increase p53 acetylation and transcription activity

**Figure 5:**
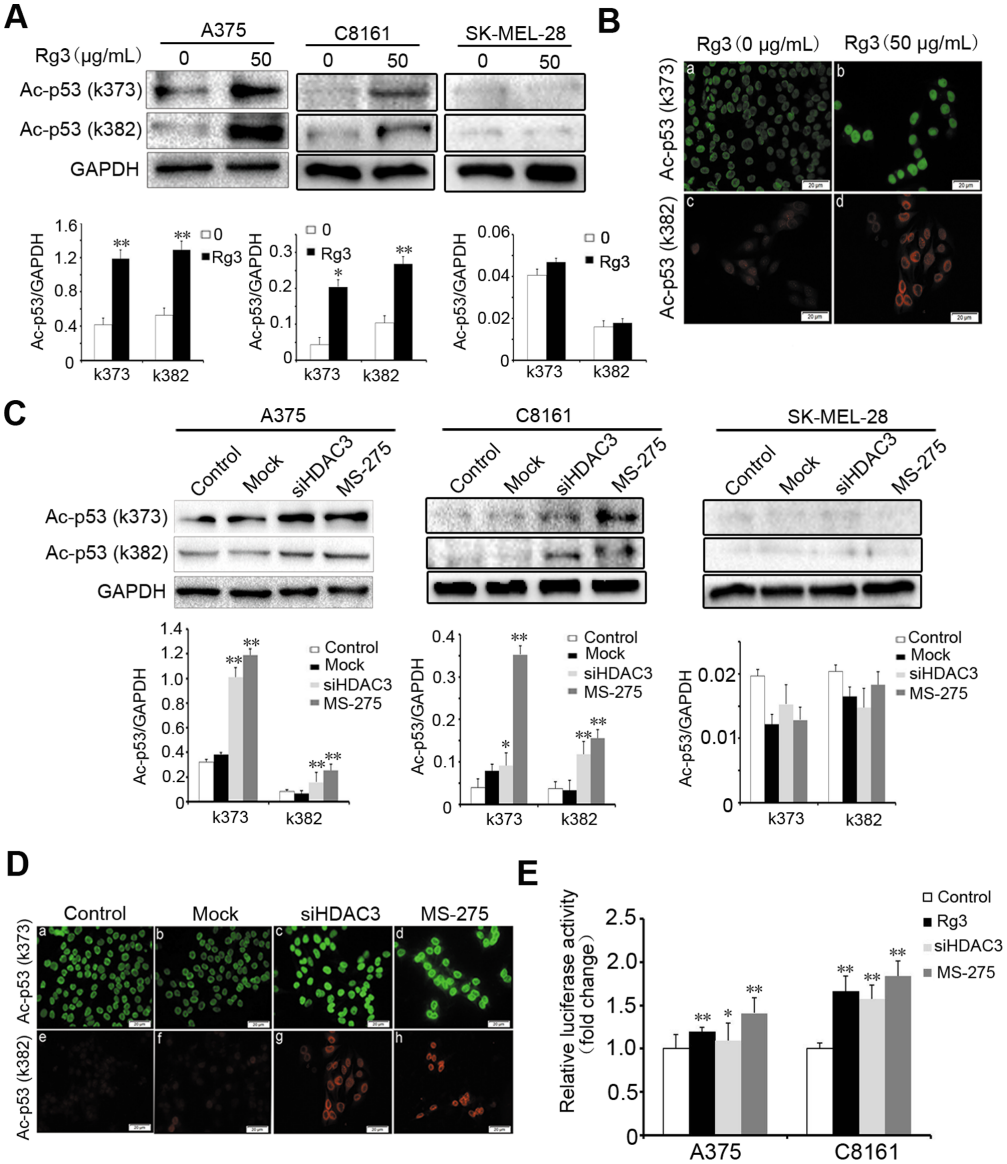
Rg3 and down-regulating HDAC3 expression increase p53 acetylation and transcription activity. Cells were treated with Rg3 (0, 50 µg/mL) for 24 h. (A) Western blotting analysis of the expression levels of Ac-p53 (k373/382) in A375, C8161 and SK-MEL-28 cells. (B) Immunofluorescent staining analysis of expression levels of Ac-p53 (k373/382) in A375 cells. Cells were transfected with HDAC3 siRNA (48 h) or exposed to MS-275 (10 µM) for 24 h. (C) Western blotting analysis the expression levels of Ac-p53 (k373/382) in A375, C8161 and SK-MEL-28 cells. (D) Immunofluorescent staining analysis the expression levels of Ac-p53 (k373/382) in A375 cells. Control: untransfected cells; Mock: cells transfected with vector; siHDAC3: cells transfected with HDAC3 siRNA. Bar = 20 µm (magnification, ×400). GAPDH was used as an internal control. The statistical analyses for Western blotting results were shown. (E) A375 and C8161 cells were transfected with p53-luciferase reporter plasmid. After 24 h, the cells were treated with Rg3 (50 µg/mL) or MS-275 (10 µM) for 24 h, or transfection with siHDAC3 for 24 h. Luciferase activity was measured in cell lysates. Data were presented as the mean±S.D. of three independent experiments. *, *p*<0.05; **, *p*<0.01.

To determine whether Rg3-mediated induction of the acetylation of p53 protein level is p53 dependent, A375, C8161 (p53 wt) and SK-MEL-28 (p53 mutant) cells were treated with Rg3 (50 µg/mL) for 24 h. Western blotting analysis showed that Rg3 increased the acetylation of p53 on lysine k373 and k382 in p53-wild type A375 and C8161 cells, but not in p53-mutated SK-MEL-28 cells (**Fig. 5A**). Immunofluorescent staining of Ac-p53 (k373/k382) in A375 cells further confirmed these results (**Fig. 5B**). Next, we tested the effects of knocking down or inhibiting HDAC3 by MS-275 (10 µM) on the acetylation of p53 on lysine k373 and k382. Western blotting analysis showed that down-regulating HDAC3 by siRNA or MS-275 led to a significant increase in the amount of acetylated p53 on lysine k373 and k382 in A375 and C8161 cells, but not in SK-MEL-28 cells (**Fig. 5C**). Immunofluorescent staining in A375 cells showed similar results (**Fig. 5D**). To further investigated the effects of Rg3, siHDAC3, and MS-275 on p53 transcription activity, p53-luciferase reporter plasmid was transfected into A375 and C8161 cells, and the luciferase activity was measured. As shown in **Fig. 5E**, treatment with Rg3, MS-275, or transfection with siHDAC3 significantly increased p53 transcription activity (*p*<0.05). These results suggest that down-regulation of HDAC3 increases p53 acetylation and transcription activity, and this appears to be critical for Rg3 inhibition of melanoma cell proliferation.

### Down-regulation of HDAC3 by Rg3 alters cell cycle protein expression

**Figure 6:**
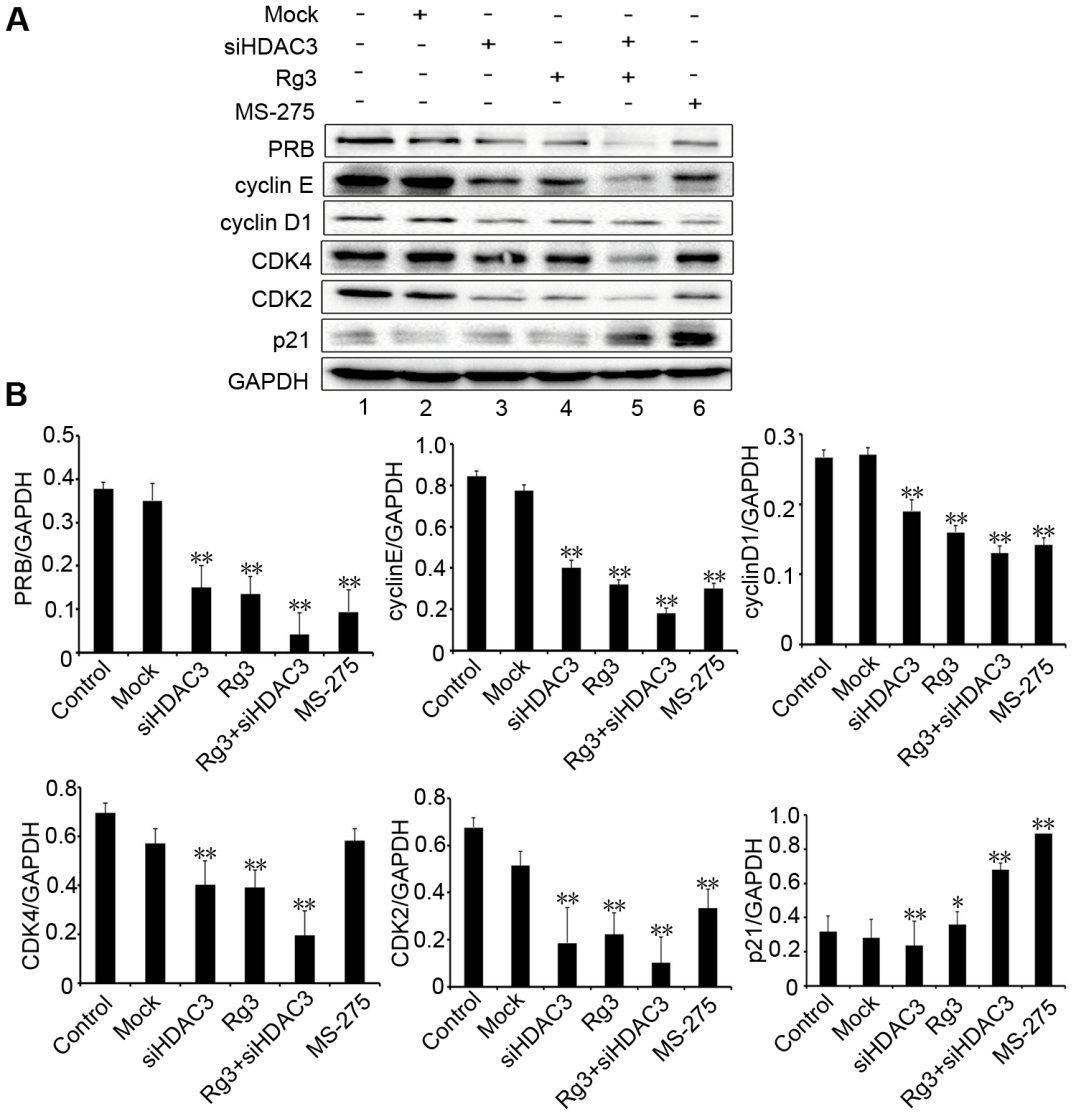
Down-regulation of HDAC3 by Rg3 alters cell cycle protein expression. A375 cells were treated with HDAC3 siRNA, Rg3 (50 µg/mL), MS-275 (10 µM), or HDAC3 siRNA transfection followed by Rg3 treatment. Control: untransfected cells; Mock: cells transfected with vector. (A) Western blotting analysis of expression levels of PRB, cyclin E, cyclin D1, CDK4, CDK2 and p21. GAPDH was used as an internal control. (B) The statistical analyses for Western blotting results were shown. Data were presented as the mean±S.D. of three independent experiments. *, *p<*0.05; **, *p<*0.01.

p53 acetylation plays an important role in the regulation of the cell cycle. As demonstrated in this study, Rg3 inhibits A375 cell proliferation by increasing the percentage of cells in G0/G1 phases, which was correlated with a decrease in HDAC3 expression, and an increase in p53 acetylation and transcription activity. We hypothesized that alterations of cell cycle protein was involved. Western blotting analysis revealed that Rg3, siHDAC3, and MS-275 reduced the expression of PRB, cyclin E, cyclin D1, CDK2, CDK4, and increased the expression of p21 in A375 cells (**Fig. 6A**). Moreover, treatment with combination of Rg3 and siHDAC3 (**Fig. 6A** **, lane 5**) was more effective in regulating the expression of cell cycle proteins than treatment with Rg3, siHDAC3, or MS-275 alone (**Fig. 6A** **, lane 5 versus lane 4, 3, 6**). These results suggest that Rg3 induced decrease in HDAC3 and increase in p53 acetylation arrest cell cycle through regulating the expression of cell cycle proteins.

### Rg3 inhibits the growth of melanoma xenograft tumors *in vivo*

**Figure 7:**
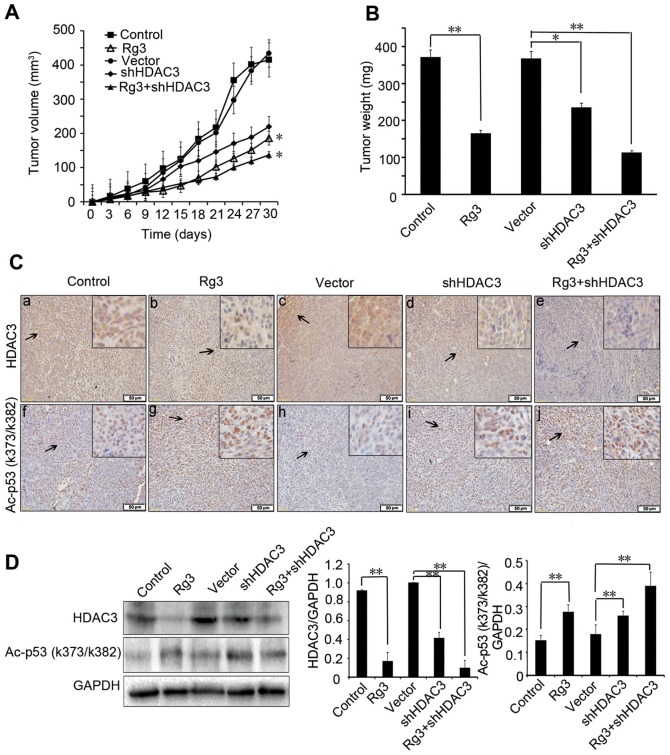
Rg3 inhibits the growth of melanoma xenograft tumors *in vivo*. A375 cells xenografted nude mice were injected with vehicle or vector (control), Rg3 (20 mg/kg), shHDAC3 (150 µg DNA/mouse) or Rg3 (20 mg/kg) combined with shHDAC3 (150 µg DNA/mouse). The animal experiment was carried out for 28 days. (A) Tumor volumes after treatment with Rg3, shHDAC3 or in combination with shHDAC3 in xenografts model (n = 6 per group). (B) Tumor weight. Group data analyses were shown. (C) Immunohistochemical staining of HDAC3 and Ac-p53 (k373/382) expression in xenograft tumor tissues. Bar = 50 µm (magnification, × 200). Insets showed enlarged views (magnification, × 400). (D) Western blotting analysis of expression levels of HDAC3 and Ac-p53 (k373/382) in lysates from the tumors in differently treated mice. GAPDH was used as an internal control. The statistical analyses for Western blotting results were shown. Data were presented as the mean±S.D. of three independent experiments. *, *p<*0.05, **, *p<*0.01.

To further investigate the inhibitory effect of Rg3 on tumor growth *in vivo*, A375 cells were inoculated into nude mice. The tumor volume and tumor weight were measured and analyzed on day 28. Compared to the untreated control or vector treatment group, as shown in **Fig. 7A**, the Rg3 treatment group significantly reduced xenograft tumor volume by 55.65% (*p<*0.05), and the shHDAC3 treatment group reduced tumor volume by 38.50% (*p<*0.05). Moreover, treatment with combination of Rg3 and shHDAC3 inhibited tumor volume by 63.69% (*p<*0.05). The tumor weight in Rg3 treatment group (0.165±0.065 g) was less than that of the untreated control group (0.371±0.117 g) (*p*<0.01) and vector group (0.368±0.085 g) (*p<*0.05). The tumor weight in Rg3 combined with shHDAC3 treatment group (0.113±0.062 g) was less than that of the Rg3 (0.165±0.065 g) or shHDAC3 group (0.235±0.085 g) (*p<*0.01) (**Fig. 7B**). To elucidate the mechanism by which Rg3 reduced tumor growth, expressions of HDAC3 and Ac-p53 (k373/k382) were examined by immunohistochemical staining. Results showed that the Rg3 treatment and Rg3 combined with shHDAC3 treatment groups had lower expression of HDAC3 and higher expression of Ac-p53 (k373/k382), when compared to those of untreated control and vector group (**Fig. 7C**). These observations were further confirmed by Western blotting (**Fig. 7D**). These results indicate that Rg3 has a strong antiproliferative effect on melanoma *in vivo*.

## Discussion

In this study, we demonstrated that Rg3 could inhibit the proliferation of human melanoma cells both *in vitro* and *in vivo.* We found that Rg3 inhibited the growth of melanoma cells in a time- and dose-dependent manner. Furthermore, Rg3 significantly reduced tumor volume and weight when compared with the control groups in A375 xenograft nude mice models. Our results indicate that Rg3 is a potential drug for the treatment of melanoma. Although several drugs have been approved for the treatment of metastatic melanoma, these treatment options are still lacking indurable responses. Dacarbazine is a conventional chemotherapeutic drug with a low response rate (11% to 25%) for metastatic melanoma [3]. High-dose of interleukin-2 (IL-2) increases relapse-free survival, but a low response rate and significant toxicities precludes its applications in many cases of melanoma [28]. The response rate is also limited and toxicity is prominent for current available targeted therapeutic agents, such as vemurafenib and ipilimumab [4], [5], [29]. Hence, it is the utmost importance to explore novel anti-melanoma drugs with high efficiency and low toxicity. Rg3 has been shown to be a beneficial adjunct to chemotherapy, and it has been reported to enhance immunity, reduce fatigue, and improve the quality of life in patients with non-small cell lung cancer [26]. Rg3 has also been shown to enhance the chemotherapeutics susceptibility of colon cancer cells, when used in combination with docetaxel or cisplatin [30], [31]. Combined with the results of this study, we predict that Rg3 may not only inhibit the proliferation of melanoma, but may also serve as an adjunctive therapy when combined with other melanoma therapies.

The antitumor mechanisms of Rg3 have been reported in many cancers, but its regulatory mechanism on HDACs is unclear. Rg3 has been shown to exert high antitumor activity by inhibiting tumor cell proliferation, angiogenesis, and invasion in prostate and lung cancers [25], [32], [33]. In hepatic and gastric cancers, Rg3 has been demonstrated to stimulate tumor apoptosis [23], [24]. In current study, we demonstrated that the antiproliferative ability of Rg3 was related to a down-regulation of HDAC3 expression and up-regulation of the acetylation of non-histone protein p53 both *in vitro* and *in vivo*. Rg3 could down-regulate the expression of HDAC3 in a time- and dose-dependent manner. It was previously reported that knockdown of HDAC3 decreased estrogen-dependent MCF-7 breast cancer cell proliferation [34]. siRNA mediated knockdown of HDAC3 reduced cell proliferation with high acetylation of Dleu2/miR-15a/16-1 in the promoter region and up-regulation of miR-15a/16-1 expression in lung cancer [35]. Our data suggests that knockdown of HDAC3 (with HDAC3 siRNA) significantly inhibited A375 and C8161 melanoma cells proliferation, and the similar results were also found in MS-275 treatment cells. The potent HDAC3 enzyme inhibitor MS-275 may regulate the expression of HDAC family members directly or indirectly. It is reported that MS-275 directly destabilizes DNMT1 by enhancing its ubiquitination and acetylation of DNMT1. The functional importance of DNMT1 acetylation is to inhibit HDAC activity and expression [36]. Shen et al reported that MS-275 induced STAT3 acetylation, which may affect the transcription and down-regulate the expression of HDACs [37]. In this study, we showed that Rg3 in combination with HDAC3 shRNA significantly inhibited melanoma growth, reduced xenograft melanoma weight and volume in melanoma mouse models, as compared to the group treated with Rg3 alone. Our results suggest that down-regulation of HDAC3 is a potential therapeutic strategy to inhibit the melanoma cell proliferation. Moreover, combination therapy of Rg3 with HDAC3 siRNA/shRNA seems to synergistically enhance anticancer effect.

p53, as a tumor suppressor, plays multiple roles in the inhibition of cell proliferation, cell cycle arrest, and DNA damage response. The activity and stability of p53 are regulated by multiple post-translational modifications, such as acetylation, phosphorylation, and ubiquitination [38]. p53 acetylation is important in promoting stabilization, enhancing the transcriptional activity, and inhibiting Human Double Minute 2 (HDM2) complexes formation [39]. Several reports showed that induced p53 acetylation on lysine k373/382 significantly increased p53 activity and p21 expression [40], [41]. p53 acetylation at either lys373 or lys382 blocks the MDM2-mediated ubiquitination of p53, which increases the steady state level of acetylated proteins [41]–[43]. In our study, we found that Rg3, HDAC3 siRNA, or MS-275 could up-regulate p53 acetylation on lysine k373/382 in a p53 dependent manner by comparing p53 wt A375, C8161 with p53 mutant SK-MEL-28 cells. Moreover, we demonstrated that Rg3, HDAC3 siRNA, or MS-275 could promote p53 transcription activity, which led to the inhibition of cell proliferation and arrest of cell cycle in the G0/G1 phase. We found that up-regulated p53 acetylation could decrease the expression of cell cycle-related proteins, such as PRB, cyclin E, cyclin D1, CDK4 and CDK2, while increase the expression of p21 in A375 melanoma cells. The results also suggest that p53 is one of the non-histone substrates of HDAC3 in melanoma cells, and HDAC3 plays an important role as a regulator of p53 acetylation and transcription activity.

Rg3 is a potential HDAC3 inhibitor in melanomas. Recently, emerging evidence has shown that HDACis can have effective antitumor activity in melanoma [44]. HDACis could sensitize metastatic melanoma to TRAIL/Apo2L-mediated immune-therapy, thereby overcoming resistance to apoptosis [45]. HDACis combined with selective BRAF inhibitors synergistically induce cell death in BRAF^V600E^ melanoma cells [46]. Combination therapy with HDACis FR901228 and suicide gene therapy enhanced anti-melanoma effects in melanoma-bearing mice [47]. Currently, there is an increasing interest in HDAC3 as a promising therapeutic target in multiple myeloma, leukemia, and gastric cancer [17], [48], [49]. In our study, we found that HDAC3 was highly expressed in human melanoma tissues compared to that of normal control. The increased expression of HDAC3 also correlated with lymph node metastasis and clinical stage of melanoma. The results suggest that HDAC3 may be a potential oncogenic factor and therapeutic target in melanoma.

In conclusion, Rg3 effectively inhibits melanoma cell growth through down-regulating HDAC3 expression, and increasing p53 acetylation and transcription activity. Our results provide the new insight into the mechanisms of Rg3 in inhibition of melanoma cell growth from epigenetic aspect. By targeting HDAC3, Rg3 is a potential novel therapeutic agent for melanoma treatment.
